# The psychological impact of the covid-19 on Tunisian healthcare workers tested positive

**DOI:** 10.1192/j.eurpsy.2022.1250

**Published:** 2022-09-01

**Authors:** A. Adouni, J. Mannai, M. Zaafrane, F. Jaballah, A. Dakhli, N. Herch, A. Fayala

**Affiliations:** Ibn Jazzar university hospital, Psychiatry, kairouan, Tunisia

**Keywords:** Covid-19, Anxiety, Depression, Healthcare professionals

## Abstract

**Introduction:**

The covid19 pandemic has led to a major health crisis and the healthcare workers, who are the first to respond, are generally the ones who pay the highest price. Their safety, both physical and psychological, should be a priority in the management of this pandemic.

**Objectives:**

We aim to assess anxiety and depression in caregivers with covid19 and to identify the many factors that may be responsible for this psychological distress.

**Methods:**

A cross-sectional survey was conducted among healthcare workers at the different health structures located in Kairouan-Tunisia from October to December 2020 within the framework of a crisis unit set up by the Ministry of Health. Through phone interviews, demographic and clinical data were collected at first, then psychological impact was evaluated using hospital anxiety and depression scale (HAD).

**Results:**

68 healthcare workers (47 females) with covid-19 were included in this study with an average age of 44.12 years: 38 nurses, 21 hospital employees and 9 doctors working at the Ibn Jazzar hospital (47%), the Aghlabite hospital (20%) and the dispensaries of Kairouan (33%). Among them, 8 had a depression score >10 and 20 had an anxiety score >10. Moreover, 90% feared contaminating their loved ones and 43% reported sleep disturbance and irritability during the containment period. The most important causes reported were the non-availability of means of protection (41,2%) and the increase in workload (64,7%).

**Conclusions:**

The prevention of the negative psychological impact of COVID-19 on the affected health professionals is essential to preserve their well-being and to ensure the continuity of care.

**Disclosure:**

No significant relationships.

